# Contributions to the cytogenetics of the Neotropical fish fauna

**DOI:** 10.3897/CompCytogen.v11i4.14713

**Published:** 2017-10-20

**Authors:** Luiz Antônio Carlos Bertollo, Marcelo de Bello Cioffi, Pedro Manoel Galetti Jr, Orlando Moreira Filho

**Affiliations:** 1 Laboratório de Citogenética de Peixes, Departamento de Genética e Evolução, Universidade Federal de São Carlos, São Carlos, SP 13565-905, Brazil; 2 Laboratório de Biodiversidade Molecular e Conservação, Departamento de Genética e Evolução, Universidade Federal de São Carlos, São Carlos, SP 13565-905, Brazil

**Keywords:** Fish cytogenetics, Neotropical species, biodiversity, sex chromosomes, B chromosomes

## Abstract

Brazilian fish cytogenetics started as early as the seventies in three pioneering research groups, located at the Universidade Estadual Paulista (UNESP, Botucatu, SP), Universidade Federal de São Carlos (UFSCar, São Carlos, SP) and Universidade de São Paulo (USP, São Paulo, SP). Investigations that have been conducted in these groups led to the discovery of a huge chromosomal and genomic biodiversity among Neotropical fishes. Besides, they also provided the expansion of this research area, with the genesis of several other South American research groups, in view of a number of dissertations and doctoral theses developed over years. The current authors were encouraged to make their thesis catalog accessible from a public source, in order to share informations on the taxa and subject matter analyzed. Some of the key contributions to evolutionary fish cytogenetics are also being highligthed.

## Introduction

About 13,000 freshwater fish species are now recognized, 50% of them living in the Neotropical region (Reis et al. 2003), which emphasizes the significant parcel of the ichthyological diversity enclosed in this particular world region. Of course, this was one of the main reasons that attracted the attention of some Brazilian researchers, fostering the investigation on cytogenetics of Neotropical fishes.

Brazilian fish cytogenetics started in the early 70s, with three pioneering research groups located at the Universidade Estadual Paulista (UNESP, Botucatu, SP), Universidade Federal de São Carlos (UFSCar, São Carlos, SP) and Universidade de São Paulo (USP, São Paulo, SP). During this time, a lot of significant evolutionary and cytotaxonomic contributions were achieved, improving the knowledge on the biodiversity inside the rich Neotropical ichthyofauna.

The development of methodological approaches was certainly a key step for obtaining good chromosomal preparations and for improving fish cytogenetics. In this sense, the direct chromosome preparation from kidney cells, adapted in our early studies since 70s and recently revised (Bertollo et al. 2015), was largely utilized over years. In addition, the progressive application of conventional banding techniques (C, AgNORs, DAPI, CMA_3_ staining), as well as more advanced methodologies combining cytogenetic and molecular procedures (chromosome mapping of DNA sequences by FISH, whole chromosome painting – WCP and comparative genomic hybridization – CGH) were essential tools in understanding the fish genome organization, particularly regarding to sex chromosome evolution and biodiversity investigations.

Although primarily and mainly devoted to freshwater species, the chromosomal analyses were also expanded to marine fishes, which is now the particular focus of some laboratories. From 1986 to now, successful biennual symposiums on fish cytogenetics are ongoing at different Brazilian regions. From some years ago, the discipline of genetics was also added to such meetings, with an expressive participation of professionals, students, as well as foreign invited researchers.

The catalogue of student theses, supervised in the Laboratory of Fish Cytogenetics of the Universidade Federal de São Carlos, comprises 42 doctoral theses and 52 master dissertations from 1981 to 2016. Informations about their corresponding students, taxa and matter subjects are available in the present communication, considering that not all the resulting data have been published. Theses/dissertations produced were assembled by taxonomic groups, according to Reis et al. (2003), regardless of their chronology. This criterion provides an overview of the different studied groups, considering that several families, genera and species have wider distribution and were subjected of more extensive investigations, being analyzed by different authors. The "taxa analyzed" item makes explicit when different populations, as well as different karyomorphs (karyotypes with distinct characteristics from each other) of a given species were investigated. The term “species group” was used for cases of specimens showing morphological similarities to a given valid species, but missing a proper taxonomic revision by the time they were studied.

Significantly, more than 20 research groups, nowadays located in different Brazilian regions, and also in Argentina, have emerged from such studies. These new researchers, along with those that have been emerged from the other pioneer laboratories, are now also engaged on fish chromosomal investigations. This was a preponderant condition for the big expansion experienced by the Brazilian fish cytogenetics.

The "Final Remarks" highlights some key contributions to fish evolutionary cytogenetics from MSc and PhD theses produced, as well as from other results that were led by our research team, some of them with significant colaborations of other national and international research groups.

Laboratory site at the Universidade Federal de São Carlos: (http://www.lcp.ufscar.br)

### Abbreviations used

**UFSCar** Universidade Federal de São Carlos

**INPA** Instituto Nacional de Pesquisas da Amazônia

**USP** Universidade de São Paulo

**UFRJ** Universidade Federal do Rio de Janeiro

**PPGGEv** Programa de Pós-Graduação em Genética Evolutiva e Biologia Molecular

**BADPI** Programa de Pós-Graduação em Biologia de Água Doce e Pesca Interior

**PPGERN** Programa de Pós-Graduação em Ecologia e Recursos Naturais

**PPGCB – Gene** Programa de Pós-Graduação em Ciências Biológicas – Genética

**PPGCB – Ecol** Programa de Pós-Graduação em Ciências Biológicas – Ecologia

**CAPES** Coordenação de Aperfeiçoamento de Pessoal do Ensino Superior

**FAPESP** Fundação de Amparo à Pesquisa do Estado de São Paulo

**CNPq** Conselho Nacional de Desenvolvimento Científico e Tecnológico

## Catalogue of MSc Dissertations and PhD Theses

**Note**: Titles of Theses and Dissertations maintain the taxonomic and/or systematic data as they were originally employed. The classification of some species and genera were later updated by review studies (Reis et al. 2003; Oliveira et al. 2011; FishBase), according to the section: **taxon/taxa analyzed**.

### I. ORDER CHARACIFORMES

**I.1. Family Characidae**

**I.1.1. Genus *Astyanax*** Baird & Girard, 1854

**I.1.1.1. MSc Dissertation by Sandra Morelli (1981)**: Aspectos citogenéticos do gênero *Astyanax* (Pisces, Characidae) / Cytogenetic studies in the genus *Astyanax* (Pisces, Characidae). **Taxa analyzed**: *A. fasciatus* (Cuvier, 1819), *A. bimaculatus* (Linnaeus, 1758), *A. schubarti* Britski, 1964, *A. scabripinnis* (Jenyns, 1842) – UFSCar / PPGERN / CNPq

**I.1.1.2. PhD Thesis by Orlando Moreira Filho (1989)**: A diversidade no complexo *scabripinnis* (Pisces, Characidae, Tetragonopterinae). Análises citogenéticas e morfológicas. / Diversity investigation in the *scabripinnis* complex (Pisces, Characidae, Tetragonopterinae). Cytogenetic and morphological analyses. **Taxon analyzed**: *A. scabripinnis* – UFSCar / PPGERN

**I.1.1.3. MSc Dissertation by Heloisa Helena Paganelli (1990)**: A variabilidade cromossômica no gênero *Astyanax* (Pisces, Characidae) e seu significado para a sistemática e evolução do grupo / Chromosomal variability in the genus *Astyanax* (Pisces, Characidae) and its significance for the systematics and evolution of the group. **Taxa analyzed**: *A. bimaculatus, A. fasciatus, A. schubarti, A. taeniatus* (Jenyns, 1842) -UFSCar / PPGERN

**I.1.1.4. MSc Dissertation by Álvaro José Justi (1993)**: Caracterização cariotípica de populações de *Astyanax fasciatus* (Characidae) de bacias hidrográficas distintas / Karyotype characterization of *Astyanax fasciatus* populations (Characidae) from different river basins. **Taxon analyzed**: *A. fasciatus* – UFSCar / PPGGEv / FAPESP

**I.1.1.5. MSc Dissertation by Vera Elisa Vicente (1994)**: Estudo do cromossomo B em três populações de *Astyanax scabripinnis* (Characidae) / B chromosome analysis in three *Astyanax scabripinnis* populations (Characidae). **Taxon analyzed**: *A. scabripinnis* – UFSCar / PPGGEv / FAPESP

**I.1.1.6. MSc Dissertation by Issakar Lima Souza (1996)**: Estudos citogenéticos em populações de *Astyanax scabripinnis* (Characidae) pertencentes a dois riachos de diferentes bacias do Sudeste Brasileiro / Cytogenetic studies in populations of *Astyanax scabripinnis* (Characidae) from two different river basins of Southeastern Brazil.**Taxon analyzed**: *A. scabripinnis* – UFSCar / PPGGEv / FAPESP

**I.1.1.7. PhD Thesis by Carlos Alberto Mestriner (1997)**: Caracterização molecular e citológica do DNA repetitivo de *Astyanax scabripinnis* (Pisces, Characidae) portador de cromossomos supranumerários / Molecular and cytological characterization of repetitive DNAs in *Astyanax scabripinnis* (Pisces, Characidae) carrying supernumerary chromosomes. **Taxon analyzed**: *A. scabripinnis* – UFSCar / PPGGEv / FAPESP

**I.1.1.8. MSc Dissertation by María Pía Heras (1998)**: Estudos citogenéticos em *Astyanax fasciatus* (Characidae) de alguns rios do Brasil / Cytogenetic studies in *Astyanax fasciatus* (Characidae) from some Brazilian rivers. **Taxon analyzed**: *A. fasciatus* populations – UFSCar / PPGGEv / CNPq

**I.1.1.9. MSc Dissertation by Daniela Morilha Néo (1999)**: Distribuição dos cromossomos B presentes em *Astyanax scabripinnis* (Characidae) ao longo do Ribeirão Grande na região de Campos do Jordão–SP / B chromosomes distribution in *Astyanax scabripinnis* (Characidae) along the Grande Stream, Campos do Jordão region–SP. **Taxon analyzed**: *A. scabripinnis* – UFSCar / PPGGEv / FAPESP

**I.1.1.10. PhD Thesis by Dagmar Aparecida de Marco Ferro (2000)**: Análises cariotípicas dos cromossomos B em populações de *Astyanax scabripinnis* (Pisces, Characidae) / Karyotypic analyses of B chromosomes in *Astyanax scabripinnis* populations (Pisces, Characidae). **Taxon analyzed**: *A. scabripinnis* – UFSCar / PPGGEv / CNPq

**I.1.1.11. MSc Dissertation by Monique Mantovani (2001)**: Citogenética comparativa entre populações de *Astyanax scabripinnis* (Characidae) da bacia do rio Paranapanema / Comparative cytogenetics among populations of *Astyanax scabripinnis* (Characidae) from the Paranapanema River basin. **Taxon analyzed**: *A. scabripinnis* – UFSCar / PPGGEv / FAPESP

**I.1.1.12. MSc Dissertation by Luciano Douglas dos Santos Abel (2001)**: A variabilidade do complexo de espécies *scabripinnis* (Characidae) como estratégia adaptativa. Estudo da diversidade cariotípica do grupo com ênfase em populações da bacia do rio São Francisco / The variability of the *scabripinnis* species complex (Characidae) as an adaptive strategy. Analysis of the karyotypic diversity emphasizing populations from the São Francisco River basin. **Taxon analyzed**: *A. scabripinnis* – UFSCar / PPGGEv / FAPESP

**I.1.1.13. PhD Thesis by Rubens Pazza (2005)**: Contribuição citogenética à análise da biodiversidade em *Astyanax fasciatus* (Pisces, Characidae) / Cytogenetic contribution to biodiversity analysis in *Astyanax fasciatus* (Pisces, Characidae). **Taxon analyzed**: *A. fasciatus* – UFSCar / PPGGEv / FAPESP

**I.1.1.14. PhD Thesis by Wellington Adriano Moreira (2009)**: Análise citogenética de espécies de *Astyanax* (Characiformes) na região de transposição do rio Piumhi / Cytogenetic analysis of *Astyanax* species (Characiformes) from the transposition region of the Piumhi River. **Taxa analyzed**: *A. scabripinnis*, *A. bimaculatus*, *A. lacustris* (Lütken, 1875), *A. altiparanae* Garutti & Britski, 2000 – UFSCar / PPGGEv / FAPESP

**I.1.2. Genus *Bryconamericus*** Eigenmann, 1907

**I.1.2.1. MSc Dissertation by Adriane Pinto Wasko (1996)**: Estudos citogenéticos no gênero *Bryconamericus* (Pisces, Characidae). Uma abordagem citotaxonômica-evolutiva / Cytogenetic studies in the *Bryconamericus* genus (Pisces, Characidae). A cytotaxonomic-evolutionary approach. **Taxa analyzed**: *Bryconamericus* sp A-E, *Piabina argentea* Reinhardt, 1867 – UFSCar / PPGGEv / CNPq

**I.1.3. Genus *Moenkhausia*** Eigenmann, 1903

**I.1.3.1. MSc Dissertation by Elisangela Santana de Oliveira Dantas (2002)**: Estudos citogenéticos entre três espécies de *Moenkhausia* (Characidae, Tetragonopterinae) de localidades diferentes / Cytogenetic studies among three *Moenkhausia* species (Characidae, Tetragonopterinae), from different localities **Taxa analyzed**: *Moenkhausia sanctae filomenae* (Steindachner,1907), *M. intermedia* Eigenmann, 1908, *Moenkhausia* sp. – UFSCar / PPGGEv / FAPESP

**I.1.4. Subfamily Bryconinae**

**I.1.4.1. Genus *Brycon*** Müller & Troschel, 1844

**I.1.4.1.1. MSc Dissertation by Vladimir Pavan Margarido (1995)**: Uma contribuição à citogenética de Bryconinae (Characiformes, Characidae) / A contribution to Bryconinae cytogenetics (Characiformes, Characidae). **Taxa analyzed**: *Brycon brevicauda* Günther, 1864, *B. lundi* Lütken, 1875, *B. orbignyanus* (Valenciennes,1850), *B. microlepis* Perugia, 1897), *B. cephalus* (Günther, 1869), *B. insignis* Steindachner, 1877, *Brycon* sp.- UFSCar / PPGGEv / CAPES

**I.1.4.1.2. PhD Thesis by Adriane Pinto Wasko (2000)**: Marcadores cromossômicos e moleculares no gênero *Brycon* (Characidae): uma contribuição à biologia evolutiva e à conservação biológica destes peixes / Chromosomal and molecular markers in the genus *Brycon* (Characidae): a contribution to its evolutionary and conservation biology. **Taxa analyzed**: *Brycon lundii*, *B. orbignyanus*, *B. microlepis*, *B. cephalus*, *B. brevicauda*, *B. insignis*, *Brycon* sp. – UFSCar / PPGGEv / FAPESP/CNPq

**I.1.5. Miscelaneous groups in Characidae**

**I.1.5.1. MSc Dissertation by Ana Luiza de Brito Silva Portela (1987)**: Citogenética de peixes da subfamília Tetragonopterinae (Characidae) / Fish cytogenetics of the Tetragonopterinae subfamily (Characidae). **Taxa analyzed**: *Tetragonopterus chalceus* Spix et Agassiz, 1829, *Piabina argentea*, *Bryconamericus stramineus* Eigenmann, 1908, *Moenkhausia costae* (Steindachner,1907), *M. intermedia* Eigenmann, 1908, *Deuterodon pedri* Eigenmann, 1908 – USP / PPGCB – Gene / CAPES

**I.1.5.2. MSc Dissertation by Sandra Cristina Pfister (1997)**: Contribuição aos estudos cariotípicos da família Characidae da bacia do rio São Francisco – Três Marias (MG) / A contribution to the karyotypic studies in the family Characidae from the São Francisco River basin – Três Marias (MG).**Taxa analyzed**: *Roeboides xenodon* (Reinhardt, 1851), *Orthospinus franciscensis* (Eigenmann, 1914), *Bryconops affinis* (Günther, 1864), *Hemigrammus marginatus* Ellis, 1911, *Moenkhausia costae* – UFSCar / PPGGEv / CNPq

**I.1.5.3. PhD Thesis by Issakar Lima Souza (2003)**: rDNAs nucleares e bandamentos cromossômicos em Salmininae e *Astyanax scabripinnis* (Characidae) / Nuclear rDNAs and chromosome banding in Salmininae and *Astyanax scabripinnis* (Characidae). **Taxa analyzed**: *A. scabripinnis*, *Salminus brasiliensis* (Cuvier, 1816), *S. hilarii* Valenciennes, 1850 – UFSCar / PPGGEv / CNPq / FAPESP

**I.1.5.4. MSc Dissertation by Wellington Adriano Moreira Peres (2005)**: Análise da diversidade cariotípica de Characidae da bacia do São Francisco / Analysis on karyotypic diversity of Characidae fishes from the São Francisco River basin. **Taxa analyzed**: *Orthospinus franciscensis*, *Serrapinnus heterodon* (Eigenmann, 1915), *S. piaba* (Lütken,1875), *Astyanax fasciatus*, *A. bimaculatus*, *Haseamania nana* (Lütken, 1875), *Piabina argentea* – UFSCar / PPGGEv / FAPESP

**I.2. Family Prochilodontidae**

**I.2.1. Genus *Prochilodus*** Agassiz, in Spix et Agassiz, 1829

**I.2.1.1. MSc Dissertation by Erica Pauls (1981)**: Evidências de um sistema de cromossomos supranumerários em *Prochilodus scrofa* Steindachner, 1881 (Pisces, Prochilodontidae) / Evidences for a supernumerary chromosome system in *Prochilodus scrofa* Steindachner, 1881 (Pisces, Prochilodontidae). **Taxon analyzed**: *Prochilodus lineatus* (Valenciennes, 1836), cited as *P. scrofa* – UFSCar / PPGERN / CNPq

**I.2.1.2. PhD Thesis by Erica Pauls (1985)**: Considerações sobre evolução cromossômica e sistema de cromossomos supranumerários em espécies do gênero *Prochilodus* (Pisces, Prochilodontidae) / Considerations on chromosomal evolution and supernumerary chromosome systems in *Prochilodus* species (Pisces, Prochilodontidae). **Taxa analyzed**: *P. lineatus* (cited as *P. scrofa*), *P. vimboides* Kner, 1859, *P. brevis* Steindacher, 1875 (cited as *P. cearensis* Steindachner, 1911), *P. argenteus* Agassiz, 1829, *P. margravii* (rejected by ICZN; under synonymy of *P. argenteus*), *P. costatus* Valenciennes, 1850 (cited as *P. affinis* Lütken, 1875, *P. nigricans* Agassiz, 1829 – UFSCar / PPGERN / CNPq

**I.2.1.3. MSc Dissertation by Zélia Isabel Cavallaro (1992)**: Estudos comparativos sobre os cromossomos B de *Prochilodus scrofa* Steindachner, 1881 (Pisces, Prochilodontidae) / Comparative studies on B chromosomes of *Prochilodus scrofa* Steindachner, 1881 (Pisces, Prochilodontidae). **Taxon analyzed**: *P. lineatus*, cited as *P. scrofa* – UFSCar / PPGERN / CAPES

**I.2.1.4. PhD Thesis by Terumi Hatanaka (2000)**: Marcadores cromossômicos e moleculares no peixe *Prochilodus marggravii*: uma espécie de interesse econômico no rio São Francisco / Chromosomal and molecular markers in *Prochilodus marggravii*, a fish species with economic significance from the São Francisco River.**Taxon analyzed**: *P. argenteus* (cited as *P. margravii*: rejected by ICZN)- UFSCar / PPGGEv / FAPESP

**I.2.1.5. PhD Thesis by Célia Maria de Jesus (2001)**: Caracterização de sequências repetitivas no genoma de *Prochilodus lineatus* (Prochilodontidae) portador de cromossomos B / Characterization of repetitive sequences in the genome of *Prochilodus lineatus* (Prochilodontidae) carrying B chromosomes. **Taxon analyzed**: *P. lineatus* – UFSCar / PPGGEv / CNPq / FAPESP

**I.3. Family Parodontidae**

**I.3.1. MSc Dissertation by Orlando Moreira Filho (1983)**: Estudos na família Parodontidae (Pisces, Characiformes – cited as Cypriniformes) da bacia do rio Passa-Cinco (SP): aspectos citogenéticos e considerações correlatas / Studies in Parodontidae species (Pisces, Characiformes) from the Passa-Cinco River Basin (SP): cytogenetic and correlated considerations.**Taxa analyzed**: *Apareiodon affinis* (Steindachner, 1879), *A. ibitiensis* Campos, 1944, *A. piracicabae* (Eigenmann, 1907), *Parodon nasus* Kner, 1859 (cited as *P. tortuosus* Eigenmann & Norris, 1900) – UFSCar / PPGERN

**I.3.2. MSc Dissertation by Célia Maria de Jesus (1996)**: Contribuição aos estudos citogenéticos na família Parodontidae (Characiformes) / Contribution to cytogenetic studies in the family Parodontidae (Characiformes). **Taxa analyzed**: *Parodon nasus* (cited as *P. tortuosus*), *P. pongonensis* (Allen, 1942) (cited as *Parodon* sp), *Apareiodon affinis, A. ibitiensis, A. piracicabae, A.vitattus* Garavello, 1977- UFSCar / PPGGEv / FAPESP

**I.3.3. PhD Thesis by Vera Elisa Vicente (2001)**: Estudos citogenéticos e moleculares em *Parodon hilarii* e correlações com outras espécies da família Parodontidae (Characiformes) / Cytogenetic and molecular studies in *Parodon hilarii* and correlations with other Parodontidae species. **Taxa analyzed**: *P. hilarii* Reinhardt, 1866, *P. nasus* (cited as *P. tortuosus*) – UFSCar / PPGGEv / CNPq

**I.3.4. MSc Dissertation by Elisangela Bellafronte da Silva (2004)**: Estudos citogenéticos comparativos em espécies do gênero *Parodon* (Parodontidae) / Comparative cytogenetics in *Parodon* species (Parodontidae).**Taxon analyzed**: *P. nasus* – UFSCar / PPGGEv / CNPq

**I.3.5. PhD Thesis by Josiane Baccarin Traldi (2015)**: Investigação do papel dos DNAs repetitivos na evolução cromossômica de espécies de *Apareiodon* (Characiformes, Parodontidae) / Investigation on the role of repetitive DNAs in the chromosomal evolution of *Apareiodon* species (Characiformes, Parodontidae). **Taxa analyzed**: *A. cavalcante* Pavanelli & Britski, 2003, *A. machrisi* Travassos, 1957, *A. argenteus* Pavanelli & Britski, 2003, *A. davisi* Fowler, 1941, *Apareiodon* sp. 1, *Apareiodon* sp. 2 – UFSCar / PPGGEv / FAPESP

**I.4. Family Erythrinidae**

**I.4.1. MSc Dissertation by Lucia Giuliano Caetano (1986)**: Estudo citogenético em *Hoplerythrinus unitaeniatus* (Pisces, Erythrinidae) de diferentes bacias hidrográficas brasileiras / Cytogenetic studies in *Hoplerythrinus unitaeniatus* (Pisces, Erythrinidae) from different Brazilian river basins. **Taxon analyzed**: *H. unitaeniatus* (Agassiz, 1829) – UFSCar / PPGERN / CNPq

**I.4.2. MSc Dissertation by Jorge Abdala Dergam dos Santos (1989)**: O cariótipo de *Hoplias malabaricus* em populações da bacia do São Francisco e do Alto Paraná. Considerações citotaxonômicas / The karyotype of *Hoplias malabaricus* populations from the São Francisco and High Paraná River basins. Cytotaxomic considerations. **Taxon analyzed**: *H. malabaricus* (Block, 1794) karyomorphs D, F – USP / PPGCB – Gene / CAPES

**I.4.3. PhD Thesis by Sandra Morelli (1998)**: Citogenética evolutiva em espécies do gênero *Hoplias*, grupo *H. lacerdae*. Macroestrutura cariotípica, heterocromatina constitutiva e regiões organizadoras de nucléolos / Evolutionary cytogenetics in *Hoplias lacerdae* species group. Karyotype macrostructure, constitutive heterochromatin and nucleolus organizing regions. **Taxon analyzed**: *H. lacerdae* Miranda Ribeiro, 1908 species group – UFSCar / PPGGEv / CNPq

**I.4.4. PhD Thesis by Guassenir Gonçalves Born (2000)**: Estudo da diversidade cariotípica no grupo *Hoplias malabaricus* (Pisces, Erythrinidae). Cariótipo 2n=42 / Study on the karyotypic diversity in the *Hoplias malabaricus* species group (Pisces, Erythrinidae). The karyotype 2n=42. **Taxon analyzed**: *H. malabaricus* karyomorphs A, B – UFSCar / PPGGEv / CAPES

**I.4.5. MSc Dissertation by Débora Diniz Bezerra (2002)**: Estudos citogenéticos populacionais em *Hoplerythrinus unitaeniatus* (Pisces, Erythrinidae). Análise da biodiversidade / Population cytogenetic studies in *Hoplerythrinus unitaeniatus* (Pisces, Erythrinidae). Biodiversity analysis. **Taxon analyzed**: *H. unitaeniatus* – UFSCar / PPGGEv / CNPq

**I.4.6. MSc Dissertation by Marcelo Ricardo Vicari (2003)**: Citogenética comparativa de *Hoplias malabaricus* (Pisces, Erythrinidae). Estudos em região divisora de águas das bacias dos rios Tibagi, Iguaçu, Ivaí e Ribeira (Ponta Grossa, PR) / Comparative cytogenetics of *Hoplias malabaricus* (Pisces, Erythrinidae). Studies in the water divisor region of the Tibagi, Iguaçu, Ivaí and Ribeira Rivers (Ponta Grossa, PR). **Taxon analyzed**: *H. malabaricus* – UFSCar / PPGGEv / FAPESP

**I.4.7. MSc Dissertation by Marcelo de Bello Cioffi (2010)**: Marcadores cromossômicos em *Hoplias malabaricus* (Characiformes, Erythrinidae). Citogenética comparativa entre cariomorfos / Chromosome markers in *Hoplias malabaricus* (Characiformes, Erythrinidae. Comparative cytogenetics among karyomorphs. **Taxon analyzed**: *H. malabaricus* karyomorphs A, B, C, D – UFSCar / PPGGEv / FAPESP

**I.4.8. MSc Dissertation by Daniel Rodrigues Blanco (2010)**: Caracterização citogenética em populações alopátricas do gênero *Hoplias*, com enfoque nos grupos *malabaricus* e *lacerdae* / Cytogenetic characterization of allopatric populations of the *Hoplias* genus, focusing on the *malabaricus* and *lacerdae* groups. **Taxa analyzed**: *H. malabaricus*, *H. aimara* (Valenciennes, 1847), *H. intermedius* (Günther, 1864) – UFSCar / PPGGEv / FAPESP

**I.4.9. PhD Thesis by Marcelo de Bello Cioffi (2011)**: Evolução cromossômica na família Erythrinidae. Mapeamento citogenético de DNAs repetitivos e microdissecção de cromossomos sexuais / Chromosome evolution in the Erythrinidae family. Cytogenetic mapping of repetitive DNAs and microdissection of sex chromosomes. **Taxa analyzed**: *Hoplias malabaricus* karyomorphs A, B, C, D, *Erythrinus erythrinus* (Bloch & Schneider, 1801) karyomorphs A, D – UFSCar / PPGGEv / FAPESP

**I.4.10. MSc Dissertation by Nícolas Fernandes Martins (2013)**: Diferenciação cromossômica em *Erythrinus erythrinus* (Characiformes, Erythrinidae) / Chromosomal differentiation in *Erythrinus erythrinus* (Characiformes, Erythrinidae). **Taxon analyzed**: *E. erythrinus* karyomorphs A, C – UFSCar / PPGGEv / CAPES

**I.4.11. MSc Dissertation by Juliana de Fátima Martinez (2014)**: *Hoplerythrinus unitaeniatus* (Characiformes, Erythrinidae): um complexo de espécies. Estudos citogenéticos e moleculares / *Hoplerythrinus unitaeniatus* (Characiformes, Erythrinidae): a species complex. Cytogenetic and molecular analyses. **Taxon analyzed**: *H. unitaeniatus* – UFSCar / PPGGEv / FAPESP

**I.4.12. MSc Dissertation by Ezequiel Aguiar de Oliveira (2015)**: Evolução cromossômica em peixes da família Erythrinidae (Characiformes). Citogenética comparativa entre espécies do gênero *Hoplias* / Chromosome evolution in the fish family Erythrinidae (Characiformes). Comparative cytogenetics among *Hoplias* species. **Taxa analyzed**: *H. aimara*, *H. brasiliensis* (Agassiz, 1829), *H. lacerdae*, *H. intermedius* – UFSCar / PPGGEv / CAPES

**I.5. Family Serrasalmidae** (Former Serrasalminae, Characidae)

**I.5.1. MSc Dissertation by Marta Margarete Cestari (1990)**: Diferenciação cromossômica no gênero *Serrasalmus* La Cèpede, 1803 e evolução do cariótipo em Serrasalminae (Pisces, Characidae) / Chromosomal differentiation in the genus *Serrasalmus* La Cèpede, 1803 and karyotypic evolution in Serrasalminae (Pisces, Characidae). **Taxa analyzed**: *S. spilopleura* Kner, 1858, *S. humerallis* Valenciennes, 1850, *S. brandti* (Lütken, 1875) – UFSCar / PPGERN / CNPq

**I.5.2. PhD Thesis by Marta Margarete Cestari (1996)**: Estudos citogenéticos e genético-bioquímicos do gênero *Serrasalmus* (Pisces, Serrasalminae) / Cytogenetic and genetic-biochemical studies in the genus *Serrasalmus* (Pisces, Serrasalminae).**Taxa analyzed**: *S. spilopleura*, *S. marginatus* Valenciennes, 1837 -UFSCar / PPGGEv / CAPES

**I.5.3. PhD Thesis by Jorge Ivan Rebelo Porto (1999)**: Análises cariotípicas e sequenciamento de DNA mitocondrial em populações de *Mylesinus paraschomburgkii* (Characiformes, Serrasalminae) da bacia amazônica / Karyotypic analyses and mtDNA sequencing in *Mylesinus paraschomburgkii* populations (Characiformes, Serrasalminae) from the Amazon Basin. **Taxon analyzed**: *M. paraschomburgkii* Jégu, Santos & Ferreira, 1989 – INPA/BADPI/CNPq

**I.5.4. PhD Thesis by Celeste Mutuko Nakayama (2007)**: Citogenética molecular comparativa do DNAr 18S e DNAr 5S em piranhas (Characidae, Serrasalminae) da Amazônia Central / Comparative molecular cytogenetics of the 18S and 5S rDNAs in piranhas (Characidae, Serrasalminae) from the Central Amazon. **Taxa analyzed**: *Serrasalmus altispinnis* Merchx, Jégu & Santos, 2000, *S. elongatus* Kner, 1858, *S. gouldingi* Fink & Machado-Allison, 1992, *S. rhombeus* (Linnaeus, 1766), *S. serrulatus* (Valenciennes, 1850), *S. maculatus* Kner, 1858, S. cf. rhombeus, *Pygocentrus nattereri* Kner, 1858, *Pristobrycon striolatus* (Steindachner, 1908), *Catoprion mento* (Cuvier, 1819) – UFSCar / PPGGEv / CNPq

**I.6. Family Triportheidae** (former Triportheinae, Characidae)

**I.6.1. PhD Thesis by José das Neves Falcão (1988)**: Caracterização cariotípica em peixes do gênero *Triportheus* (Teleostei, Characiformes, Characidae) / Karyotypic characterization of *Triportheus* fish (Teleostei, Characiformes, Characidae). **Taxa analyzed**: *T. signatus* (Garman, 1890), *T. angulatus* (Spix & Agassiz, 1829) (cited as *T. flavus* Cope, 1872), *T. albus* Cope, 1872, *T. culter* (Cope, 1872), *T. auritus* (Valenciennes, 1850) (cited as *T. elongatus* (Günther, 1864) – USP / PPGCB – Gene / CAPES

**I.6.2. PhD Thesis by Roberto Ferreira Artoni (1999)**: Citogenética do sistema de cromossomos sexuais ZZ/ZW no gênero *Triportheus* (Pisces, Characidae) / Cytogenetics of the ZZ/ZW sex chromosome system in the genus *Triportheus* (Pisces, Characidae). **Taxa analyzed**: Triportheus cf. auritus, cited as T. cf. elongatus, *T. guentheri* (Garman, 1890), *T. nematurus* (Kner, 1858) (cited as *T. paranensis* (Günther, 1874) – UFSCar / PPGGEv / FAPESP

**I.6.3. PhD Thesis by Débora Diniz Bezerra** (**2007)**: Origem e diferenciação do sistema de cromossomos sexuais ZZ/ZW em *Triportheus* (Characiformes, Characidae). Citogenética, mapeamento de genes ribossomais e microdissecção cromossômica / Origin and differentiation of the ZZ/ZW sex chromosome system in *Triportheus* (Characiformes, Characidae). Cytogenetic mapping of ribosomal genes and chromosomal microdissection. **Taxa analyzed**: *T. nematurus*, *T. guentheri*, *T. trifurcatus* (Castelnau, 1855), *T. auritus*, *T. angulatus*, *T. albus*, *Triportheus cf. signatus* – UFSCar / PPGGEv / CNPq

**I.6.4. PhD Thesis by Cássia Fernanda Yano (2016)**: Estudos evolutivos no gênero *Triportheus* (Characiformes, Triportheidae) com enfoque na diferenciação do sistema de cromossomos sexuais ZZ/ZW / Evolutionary studies in the *Triportheus* genus (Characiformes, Triportheidae) foccusing on the differentiation of the ZZ/ZW sex chromosome system. **Taxa analyzed**: *T. auritus*, *T. guentheri*, *T. albus*, *Triportheus aff. rotundatus* (Jardine, 1841), *T. nematurus*, *T. signatus*, *T. trifurcatus*, *T. pantanensis* Malabarba, 2004 – UFSCar / PPGGEv / CAPES

**I.7. Family Curimatidae**

**I.7.1. PhD Thesis by Eliana Feldberg (1990)**: Estudos citogenéticos em doze espécies de peixes da família Curimatidae (Characiformes) da Amazônia Central / Cytogenetic studies of twelve Curimatidae species (Characiformes) from the Central Amazon.**Taxa analyzed**: *Potamorhina pristigaster* (Steindachner, 1876), *P. altamazonica* (Cope, 1878), *P. latior* (Spix & Agassiz, 1829), *Curimata ocellata* (Eigenmann & Eigenmann, 1889), *C. vittata* (Kner, 1858), *C. kneri* (Steindachner, 1876), *C. cyprinoides* (Linnaeus, 1766), *Curimata* sp, *Psectrogaster rutiloides* (Kner, 1858), *Curimatella alburna* (Müller & Troschel, 1844), *C. meyeri* (Steindachner, 1882) – INPA / BADPI

**I.7.2. MSc Dissertation by Paulo Cesar Venere (1991)**: Citogenética comparativa de peixes da família Curimatidae (Characiformes) / Comparative cytogenetics of Curimatidae fish (Characiformes). **Taxa analyzed**: *Cyphocharax gilberti* (Quoy et Gaimard, 1824), *C. modestus* (Fernández-Yépez, 1948), *C. nagellii* (Steindachner, 1881), *C. vanderi* (Britski, 1980), *C. voga* (Hensel, 1870), *Cyphocharax* sp., *Steindachnerina elegans* (Steindachner, 1874), *Steindachnerina* sp., *S. insculpta* (Fernández-Yépez, 1948), *Curimatella lepidura* (Eigenmann & Eigenmann, 1889) – UFSCar / PPGERN / CAPES

**I.7.3. PhD Thesis by Rosângela Martins de Oliveira (2011)**: Citogenética clássica e molecular de três espécies de curimatídeos, com ênfase no cromossomo B de *Cyphocharax nagelli* (Characiformes, Curimatidae) / Conventional and molecular cytogenetics in three curimatid species, with emphasis on the B chromosome of *Cyphocharax nagelli* (Characiformes, Curimatidae). **Taxa analyzed**: *C. nagelli*, *C. modestus*, *Steindachnerina insculpta* – UFSCar / PPGGEv / CAPES/CNPq

**I.8. Family Crenuchidae** (former Characidiinae, Characidae)

**I.8.1. MSc Dissertation by Carlos Suetoshi Miyazawa (1991)**: Estudos citogenéticos em peixes do grupo *Characidium* (Characidiinae, Characidae), de distintas bacias hidrográficas / Cytogenetic studies in *Characidium* (Characidiinae, Characidae) species from different hydrographic basins.**Taxa analyzed**: *C. pterostictum* Gomes, 1947, Characidium cf. zebra Eigenmann, 1909, Characidium cf. lagosantense Travassos, 1947, *Characidium* sp. – UFSCar / PPGERN / CAPES

**I.9. Family Anostomidae**

**I.9.1. MSc Dissertation by Carlos Alberto Mestriner (1993)**: Análise das regiões organizadoras de nucléolo e investigação do sistema XX/XY descrito para *Leporinus lacustris* (Pisces, Anostomidae) / Analyses of the nucleolus organizer regions and investigation of the XX/XY sex system of *Leporinus lacustris* (Pisces, Anostomidae). **Taxon analyzed**: *L. lacustris* Campos, 1945 – UFSCar / PPGGEv / FAPESP

**I.9.2. MSc Dissertation by Wagner Franco Molina (1995)**: Cromossomos sexuais e polimorfismo cromossômico no gênero *Leporinus* (Pisces, Anostomidae) / Sex chromosomes and chromosomal polymorphism in the genus *Leporinus* (Pisces, Anostomidae).**Taxa analyzed**: *L. elongatus* Valenciennes, 1850, *L. obtusidens* (Valenciennes, 1836), *L. reinhardti* Lütken, 1875, Leporinus aff. elongatus – UFSCar / PPGGEv / CAPES

**I.9.3. MSc Dissertation by Cesar Martins (1997)**: Novas contribuições à citogenética de Anostomidae (Pisces, Characiformes). Citotaxonomia e filogenia no gênero *Schizodon* / New contributions to cytogenetics of Anostomidae (Pisces, Characiformes). Cytotaxonomy and phylogeny of the genus *Schizodon*. **Taxa analyzed**: *S. altoparanae* Garavello & Britski, 1990, *S. nasutus* Kner, 1858, *S. knerii* (Steindachner, 1875), *S. vittatus* (Valenciennes, 1850), *S. fasciatus* Spix & Agassiz, 1829, *S. borelli* (Boulenger, 1900), *S. isognathus* Kner, 1858, *S. intermedius* Garavello & Britski, 1990 – UFSCar / PPGGEv / CNPq

**I.9.4. MSc Dissertation by Suelen Regina Lopes Krichanã (1999)**: Contribuição ao estudo citogenético da família Anostomidae (Pisces, Characiformes) na região Amazônica / Contribution to the cytogenetics of the family Anostomidae (Pisces, Characiformes) from the Amazon region- **Taxa analyzed**: *Laemolita taeniata* (Kner, 1858), *Leporinus agassizii* Steindachner, 1876, *Leporinus cylindriformis* Borodin, 1929, *Leporinus fasciatus*, *Leporinus friderici*, *Leporinus granti* Eigenmann, 1912, *Rhythiodus microlepis*, *Schizodon fasciatus* – UFSCar / PPGGEv

**I.9.5. PhD Thesis by Cesar Martins (2000)**: Organização do DNA ribossômico 5S no genoma de peixes, com ênfase em *Leporinus* / Organization of the 5S rDNA in the fish genome, with emphasis on *Leporinus*. **Taxa analyzed**: *L. elongatus, L. obtusidens*, *L. friderici* (Block, 1794), *L. cf. elongatus*, *L. reinhardti*, *L. piau* Fowler, 1941, *L. desmotes* Fowler, 1914, *L. conirostris* Steindachner, 1875, *Schizodon altoparanae*, *S. borelli*, *S. isognathus*, *S. nasutus*, *S. knerii*, *S. vittatus* – UFSCar / PPGGEv / FAPESP

**I.9.6. PhD Thesis by Vladimir Pavan Margarido (2000)**: Uma contribuição à citogenética de Anostomidae, com ênfase na variabilidade das regiões organizadoras de nucléolos no gênero *Leporinus* (Pisces, Characiformes) / Contribution to Anostomidae cytogenetics, with emphasis on the variabilty of the nucleolar organizing regions in the genus *Leporinus* (Pisces, Characiformes). **Taxa analyzed**: *L. copelandii* Steindachner, 1875, *L. conirostris*, *L. desmotes*, *L. elongatus*, L. cf. elongatus, *L. fasciatus* (Block, 1794), *L. friderici*, *L. lacustris*, *L. macrocephalus* Garavello and Britski, 1988, *L. mormyrops* Steindachner, 1875, *L. obtusidens*, *L. octofasciatus* Steindacher, 1915, *L. piau*, *L. reinhardti*, *L. striatus Kner*, 1858, *L. taeniatus* Lütken, 1875, *L. tigrinus* Borodin, 1929 – UFSCar / PPGGEv / CNPq

**I.9.7. PhD Thesis by Cecília Teixeira de Aguilar (2001)**: Estudos citogenéticos e moleculares em populações brasileiras de *Leporellus vittatus* (Characiformes, Anostomidae) / Cytogenetic and molecular studies in Brazilian populations of *Leporellus vittatus* (Characiformes, Anostomidae). **Taxon analyzed**: *L. vittatus* (Valenciennes, 1850) – UFSCar / PPGGEv / CAPES

### II. ORDER SILURIFORMES

**II.1. Family Heptapteridae**

**II.1.1. MSc Dissertation by Alberto Sergio Fenocchio (1987)**: Polimorfismo cromossômico em *Rhamdia hilarii* (Pisces, Heptapteridae citado como Pimelodidae) / Chromosomal polymorphism in *Rhamdia hilarii* (Pisces, Heptapteridae). **Taxon analyzed**: *R. quelen* (Quoy & Gaimard, 1824) (cited as *Rhamdia hilarii* (Valenciennes, 1840) – USP / PPGCB – Gene / CNPq

**II.2. Family Loricariidae**

**II.2.1. MSc Dissertation by Roberto Ferreira Artoni (1996)**: Estudos citogenéticos na família Loricariidae, com especial ênfase no gênero *Hypostomus* Lacepede (1803) – Pisces, Siluriformes / Cytogenetic studies in the family Loricariidae, with special emphasis on the *Hypostomus* genus Lacepede (1803) – Pisces, Siluriformes. **Taxa analyzed**: *H. ancistroides* (Ihering, 1911), *H. regani* (Ihering, 1905), *H. albopunctatus* (Regan, 1908), *Hypostomus aff. auroguttatus* Kner, 1854, *Squaliforma emarginata* (Valenciennes, 1840) (cited as *Hypostomus emarginatus* Valenciennes in Cuvier et Valenciennes, 1840), *Hypostomus* sp., *Rhinelepsis aspera* Spix & Agassiz, 1829, *Liposarcus* sp., *Pogonopoma wertheimeri* (Steindachner, 1867), Panaque cf. nigrolineatus (Peters, 1877), *Hemiancistrus* sp., Sturisoma cf. nigrirostrum Fowler, 1940 -UFSCar / PPGGEv / CNPq

**II.2.2. PhD Thesis by Lúcia Giuliano-Caetano (1998)**: Polimorfismo cromossômico Robertsoniano em populações de *Rineloricaria latirostris* (Pisces, Loricariidae) / Robertsonian chromosomal polimorphism in *Rineloricaria latirostris* populations (Pisces, Loricariidae).**Taxa analyzed**: *R. rialatirostris* (Boulenger, 1900), *R. pentamaculata* Langeani & Araujo, 1994 -UFSCar / PPGGEv / CAPES

**II.2.3. MSc Dissertation by Fábio Mendes Camilo (2004)**: Estudos citogenéticos de algumas espécies de peixes da família Loricariidae pertencentes à bacia do rio Piracicaba / Cytogenetic studies in Loricariidae fish species from the Piracicaba River basin.**Taxa analyzed**: *Corumbatai acuestae* Britsky, 1997, *Liposarcus anisitsi* (Eigenmann & Kennedy, 1903), *Hypostomus albopunctatus* -UFSCar / PPGGEv

**II.2.4. PhD Thesis by Sandra Mariotto (2008)**: Estudo citogenético clássico e molecular em quinze espécies da tribo Ancistrini (Siluriformes, Loricariidae) de três bacias hidrográficas brasileiras / Conventional and molecular cytogenetic studies in 15 Ancistrini species (Siluriformes, Loricariidae) from three Brazilian hidrographic basins. **Taxa analyzed**: Ancistrus cf. dubius Eigenmann & Eigenmann, 1889, and other not identified *Ancistrus* species – UFSCar / PPGGEv / CNPq / CAPES

**II.2.5. MSc Dissertation by Ernani de Oliveira Mendes Neto (2008)**: Estudos citogenéticos em algumas espécies de Loricariidae (Teleostei, Siluriformes) da região de transposição do rio Piumhi para o rio São Francisco / Cytogenetic studies in Loricariidae species (Teleostei, Siluriformes) from the transposition region of the Piumhi River into the São Francisco River. **Taxa analyzed**: *Hypostomus regani*, *Hypostomus* sp.1, *Hypostomus* sp. 2, Rineloricaria cf. latirostris – UFSCar / PPGGEv / FAPESP

**II.2.6. PhD Thesis by Marceleia Rubert (2011)**: Estudos citogenéticos em espécies das tribos Hipostomini e Ancistrini (Loricariidae, Hypostominae) / Cytogenetic studies in Hipostomini and Ancistrini species (Loricariidae, Hypostominae). **Taxa analyzed**: *Ancistrus brevipinnis* (Regan, 1904), *A. multispinis* (Regan, 1912), *Hemiancistrus punctulatus* Cardoso & Malabarba, 1999, *Hypostomus albopunctatus*, *H. cochiodon* Kner, 1854, *H. commersoni* Valenciennes, 1836, *H. heraldoi* Zawadzki, Weber & Pavanelli, 2008, *H. hermanni* (Ihering, 1905), *H. iheringii* (Regan, 1908), *H. mutucae* Knaack, 1999, *H. nigromaculatus* (Schubart, 1964), *H. paulinus* (Ihering, 1905), H. aff. paulinus, *H. regani*, *H. strigaticeps* (Regan, 1908) – UFSCar / PPGGEv / CAPES / CNPq

**II.2.7. MSc Dissertation by Josiane Baccarin Traldi (2012)**: Citogenética comparativa em espécies de *Hypostomus* (Siluriformes, Loricariidae, Hypostominae). Contribuição da fração repetitiva do genoma para a diversidade cromossômica do grupo / Comparative cytogenetics in *Hypostomus* species (Siluriformes, Loricariidae, Hypostominae). Contribution of the repetitive genomic fraction to chromosomal diversity. **Taxa analyzed**: *H. ancistroides*, *H. iheringii*, *H. nigromaculatus*, *H. tapijara* Oyakawa, Akama & Zanata, 2005 – UFSCar / PPGGEv / FAPESP

**II.2.8. PhD Thesis by Daniel Rodrigues Blanco (2012)**: Estudos citogenéticos clássicos e moleculares em espécies do gênero *Harttia* (Siluriformes, Loricariidae), com enfoque no papel dos DNAs repetitivos na evolução cariotípica do grupo / Conventional and molecular cytogenetic studies in *Harttia* species (Siluriformes, Loricariidae), focusing on the role of repetitive DNAs in the karyotypic evolution. **Taxa analyzed**: *H. loricariformes* Steindachner, 1877, *H. longipinna* Langeani, Oyakawa & Montoya-Burgos, 2001, *H. kronei* Miranda Ribeiro, 1908, *H. gracilis* Oyakawa, 1993, *H. punctata* Rapp Py-Daniel & Oliveira, 2001, *H. torrenticola* Oyakawa, 1993, *H. carvalhoi* Miranda Ribeiro, 1939 – UFSCar / PPGGEv / FAPESP

**II.3. Family Auchenipteridae**

**II.3.1. MSc Dissertation by Roberto Laridondo Lui (2010)**: Análises comparativas citogenéticas e do DNA mitocondrial em *Parauchenipterus galeatus* Bleeker, 1862 (Siluriformes, Auchenipteridae) coletados no alto rio Paraná, no alto rio São Francisco e no rio Piumhi: um enfoque biogeográfico / Cytogenetic and mtDNA comparative analyses in *Parauchenipterus galeatus* Bleeker, 1862 (Siluriformes, Auchenipteridae) from the upper Paraná, Upper São Francisco and Piumhi Rivers: a biogeographical focus.**Taxon analyzed**: *Trachelyopterus galeatus* (Linnaeus, 1766) [cited as *P. galeatus* (Linnaeus, 1766)] – UFSCar / PPGGEv / FAPESP

**II.3.2. PhD Thesis by Roberto Laridondo Lui (2012)**: Estudos evolutivos em Auchenipteridae (Siluriformes): citogenética, DNA mitocondrial e DNA satélite / Evolutionary studies in Auchenipteridae (Siluriformes): cytogenetics, mtDNA and satellite DNA.**Taxa analyzed**: *Ageneiosus inermis* (Linnaeus, 1766), *Glanidium ribeiroi* (Haseman, 1911), *Trachelyopterus galeatus* (cited as *Parauchenipterus galeatus*), *Trachelyopterus striatulus* (Steindachner, 1877) [cited as *Parauchenipterus striatulus* (Steindachner, 1877)], *Trachelyopterus* sp., *T. neivai* (Ihering, 1930), *Tatia jaracatia* Pavanelli & Bifi, 2009- UFSCar / PPGGEv / FAPESP

### III. ORDER PERCIFORMES

**III.1. Family Cichlidae**

**III.1.1. MSc Dissertation by Eliana Feldberg (1983)**: Estudos citogenéticos em 10 espécies da família Cichlidae (Pisces, Perciformes) / Cytogenetic studies in ten Cichlidae species (Pisces, Perciformes). **Taxa analyzed**: *Astronotus ocellatus* (Agassiz, 1831), *Cichlasoma facetum* (Jenyns, 1842), *Chaetobranchopsis australe* Eigenmann & Ward, 1907, *Crenicichla lacustris* (Castelnau, 1855), *C. lepidota* Keckel,1840, *C. vittata* Heckel, 1840, *C. semifasciata* (Heckel, 1840) (cited as *Batrachops semifasciatus* Heckel, 1840), *Geophagus brasiliensis* (Quoy & Gaimard, 1824), *G. surinamensis* (Block, 1791), *Gymnogeophagus balzanii* (Perugia, 1891) -UFSCar / PPGERN / CAPES

**III.2. Family Serranidae**

**III.2.1. MSc Dissertation by Cecilia Texeira Aguilar (1993)**: Estudos citogenéticos em peixes da família Serranidae (Osteichthyes- Perciformes) ocorrentes na Baía de Guanabara – RJ / Cytogenetic studies in fishes of the family Serranidae (Osteichthyes-Perciformes) from the Guanabara Bay – RJ. **Taxa analyzed**: *Diplectrum radiale* (Quoy & Gaimard, 1824), *D. formosum* (Linnaeus, 1766), *Epinephelus marginatus* (Lowe, 1834) (cited as *Epinephelus guaza*; not of Linnaeus, 1758), *Mycteroperca rubra* (Bloch,1793), *Serranus flaviventris* (Cuvier, 1829) – UFRJ / PPGCB – Gene / CAPES

**III.3. Family Pomacentridae**

**III.3.1. PhD Thesis by Wagner Franco Molina (2000)**: Análise da diversidade genética na famíla Pomacentridae (Pisces, Perciformes), utilizando métodos combinados de citogenética, marcadores moleculares e morfometria / Analysis of the genetic diversity in the family Pomacentridae (Pisces, Perciformes), employing cytogenetic, molecular and morphometric methods. **Taxa analyzed**: *Stegastes fuscus* (Cuvier, 1830), *S. variabilis* (Castelnau, 1855), *S. leucostictus* (Müller & Troschel, 1848), *S. pictus* (Castelnau, 1855), *S. rocasensis* (Emery, 1972), *S. sanctipauli* Lubbock & Edwards, 1981, *Abudefduf saxatilis* (Linnaeus, 1758), *Chromis multilineata* (Guichenot, 1853), *C. insolata* (Cuvier, 1830), *C. flavicauda* (Günther, 1880), *Microspathodon chrysurus* (Cuvier, 1830), *Amphiprion frenatus* Brevoort, 1856 – UFSCar / PPGGEv / CAPES

### IV. ORDER OSTEOGLOSSIFORMES

**IV.1. Family Arapaimidae**

**IV.1.1. PhD Thesis by Débora Karla Marques (2003)**: Caracterização genética do pirarucu, *Arapaima gigas* (Teleostei, Arapaimidae) / Genetic characterization of the pirarucu, *Arapaima gigas* (Teleostei, Arapaimidae). **Taxon analyzed**: *A. gigas* (Schinz, 1822) – UFSCar / PPGGEv

**V. MISCELANEOUS GROUPS**

**V.1. MSc Dissertation by José das Neves Falcão (1983)**: Estudos citogenéticos em Acestrorhynchinae e Cynopotaminae (Pisces, Characidae) / Cytogenetic studies in Acestrorhynchinae and Cynopotaminae (Pisces, Characidae). **Taxa analyzed**: Order Characiformes – Family Acestrorhynchidae: *Acestrorhynchus altus* Menezes, 1969, *A. lacustris* (Lütken, 1875), Family Characidae: *Galeocharax knerii* (Steindacher, 1879), *Oligosarcus hepsetus* (Cuvier, 1829), *O. jenynsii* (Günther, 1864), *Oligosarcus* sp., *O. pintoi* Amaral Campos, 1945 (cited as *Paroligosarcus pintoi*) – USP / PPGCB – Gene / CAPES

**V.2. PhD Thesis by Mario Jorge Ignacio Brum (1994)**: A evolução cariotípica dos teleósteos marinhos e suas correlações com a filogenia deste grupo / Karyotype evolution of marine teleosts and its correlation with the phylogeny of the group. **Taxa analyzed** – Order Clupeiformes – Family Clupeidae: *Brevoortia aurea* (Spix & Agassiz, 1829); Order Perciformes–Family Haemulidae: *Orthopristis ruber* (Cuvier, 1830); Family Blenniidae: *Scartella cristata* (Linnaeus, 1758) – Order Tetraodontiformes – Family Tetraodontidae: *Sphaeroides greeleyi* Gilbert, 1900 – UFSCar / PPGGEv

**V.3. MSc Dissertation by Lilian Cristina Jorge (1995)**: Estudos citogenéticos comparativos de algumas espécies de peixes da região de Corrientes (Argentina) com as do Alto Paraná / Comparative cytogenetic studies of some fish species from the Corrientes region (Argentina) with those of the upper Paraná River basin.**Taxa analyzed** – Order Characiformes – Family Characidae: *Astyanax bimaculatus*; Family Parodontidae: *Apareiodon affinis*; Family Anostomidae: *Leporinus obtusidens*; Family Erythrinidae: *Hoplias malabaricus*, *Hoplerythrinus unitaeniatus* – UFSCar / PPGGEv / FAPESP.

**V.4. MSc Dissertation by Margareth Maria de Oliveira Correa (1995)**: Contribuição à citotaxonomia dos Scorpaeniformes (Osteichthyes-Teleostei). Estudos citogenéticos em espécies do litoral do Rio de Janeiro, Brasil / Contribution to the cytotaxonomy of Scorpaeniformes (Osteichthyes-Teloestei). Cytogenetic studies in coastal species from Rio de Janeiro, Brazil. **Taxa analyzed** – Order Scorpaeniformes–Family Dactylopteridae: *Dactylopterus volitans* (Linnaeus, 1758); Family Scorpaenidae: *Scorpaena brasiliensis* Cuvier, 1829; *S. isthmensis* Meek & Hildebrand, 1928; Family Triglidae: *Prionotus punctatus* (Block, 1793) – UFRJ / PPGCB-Ecol / CNPq

**V.5. PhD Thesis by Carlos Suetoshi Miyazawa (1997)**: Citogenética de caracídeos do rio Paraguai. Análises citotaxonômica-evolutivas e considerações biogeográficas / Cytogenetics of characids from the Paraguay River. Cytotaxonomic and evolutionary analyses and biogeographical considerations. **Taxa analyzed**: Order Characiformes – Family Acestrorhynchidae: *Acestrochynchus pantaneiro* Menezes, 1992; Family Serrasalmidae: *Metynnis maculatus* (Kner, 1858), *Myleus levis* (Eigenmann & McAtee, 1907) (cited as *Myloplus levis*); Family Characidae: *Poptella paraguayensis* (Eigenmann,1907), *Tetragonopterus argenteus* (Cuvier, 1816), *Roeboides* sp.; Astyanax cf. abramis (Jenyns, 1842), *Markiana nigripinnis* (Perugia, 1891), *Gymnocorymbus ternetzi* (Boulenger, 1895), *Moenkhausia dichroura* (Kner, 1858); Family Triportheidae: *Triportheus* sp., Family Gasteropelecidae: *Thoracocharax stellatus* (Kner, 1858) – UFSCar / PPGGEv / CAPES

**V.6. PhD Thesis by Paulo Cesar Venere (1998)**: Diversificação cariotípica em peixes do médio rio Araguaia, com ênfase em Characiformes e Siluriformes (Teleostei, Ostariophysi) / Karyotype diversification in fishes from the middle Araguaia River, with emphasis on Characiformes and Siluriformes (Teleostei, Ostariophysi). **Taxa analyzed**: Order Characiformes – Family Anostomidae: *Leporinus friderici*, *L. trifasciatus* Steindachner, 1876, *Leporinus* sp., Leporinus aff. brunneus Myers, 1950, *Laemolyta petiti* Géry, 1964; Family Prochilodontide: *Prochilodus nigricans*; Family Chilodontidae: *Caenotropus labyrinthicus* (Kner, 1858); Family Curimatidae: *Steindachnerina amazônica* (Steindachner, 1911), *S. gracilis* Vari & Williams Vari, 1989, *Curimata inornata* Vari, 1989, *Psectrogaster amazônica* Eigenmann & Eigenmann, 1889; Family Hemiodontidae: Hemiodus aff. ternetzi Myers, 1927, *H. unimaculatus* (Bloch, 1794), *Bivibranchi avelox* (Eigenmann & Myers, 1927); Family Characidae: *Roeboides* sp., *Galeocharax gulo* (Cope, 1870), *Exodon paradoxus* Müller & Troschel, 1844; Order Siluriformes – Family Doradidae: *Hassar wilderi* Kindle, 1895, *Leptodoras acipenserinus* (Günther, 1868), *Opsodoras* sp., *Rinodoras* sp.; Family Auchenipteridae: Trachelyopterus aff. galeatus (cited as Parauchenipterus aff. galeatus); Family Callichthyidae: *Megalechis thoracata* (Valenciennes, 1840), cited as *Megalechis personata* (Ranzani, 1841); Family Gymnotidae: Gymnotus aff. carapo Linnaeus, 1758 – UFSCar / PPGGEv / CAPES

**V.7. MSc Dissertation by Paulo Roberto Antunes de Mello Affonso (2000)**: Caracterização citogenética de peixes de recifes de corais das famílias Pomacanthidae e Chaetodontidae (Perciformes) / Cytogenetic characterization of coral reef fishes of the Pomacanthidae and Chaetodontidae families (Perciformes). **Taxa analyzed**: Order Perciformes – Family Pomacanthidae: *Centropigea urantonotus* Burgess, 1974, *Holocanthus ciliaris* (Linnaeus, 1758), *H. tricolor* (Block, 1795), *Pomacanthus arcuatus* (Linnaeus, 1758), *P. paru* (Block, 1787); Family Chaetodontidae: *Chaetodon striatus* Linnaeus, 1758 – UFSCar / PPGGEv / CNPq

**V.8. MSc Dissertation by Marilza Barbosa de Almeida Marques (2002)**: Estudos citogenéticos em *Conorhynchus conirostris* e *Lophiosilurus alexandri* (Siluriformes), espécies endêmicas do rio São Francisco / Cytogenetic studies in *Conorhynchus conirostris* and *Lophiosilurus alexandri* (Siluriformes), endemic species from the São Francisco River.**Taxa analyzed**: Order Siluriformes – Family Pimelodidae: *C. conirostris* (Valenciennes, 1840); Family Pseudopimelodidae: *L. alexandri* Steindachner, 1876 – UFSCar / PPGGEv

**V.9. MSc Dissertation by Karine Frehner Kavalco (2003)**: Contribuição citogenética à análise da biodiversidade da ictiofauna das nascentes do rio Paraitinga. / Cytogenetic contribution to the biodiversity analysis of the fish fauna from the headwaters of the Paraitinga River. **Taxa analyzed**: Order Siluriformes – Family Loricariidae: *Harttia loricariformes* Steindachner, 1877, *Neoplecostomus microps* (Steindachner, 1877), *Hypostomus affinis* (Steindachner, 1877), *Upsilodus* sp.; Order Characiformes – Family Characidae: *Astyanax scabripinnis*, *A. parahybae* Eigenmann, 1908, *A. intermedius* Eigenmann, 1908, *A. giton* Eigenmann, 1908, *Oligosarcus hepsetus* – UFSCar / PPGGEv / FAPESP

**V.10. PhD Thesis by Liano Centofante (2003)**: Citogenética comparativa entre ictiofaunas isoladas por um divisor de águas em regiões limítrofes de duas bacias hidrográficas na Serra da Mantiqueira / Comparative cytogenetics of fish fauna from neighboring regions of two hydrographic basins isolated by a watershed in the Serra da Mantiqueira. **Taxa analyzed**: Order Siluriformes – Family Loricariidae: *Harttia carvalhoi* Miranda Ribeiro, 1939; Family Heptapteridae: *Rhamdia* sp.; Order Characiformes – Family Characidae – *Astyanax parahybae*, *A. fasciatus*, *Hyphessobrycon anisitsi* (Eigenmann, 1907); Family Parodontidae: *Parodon nasus* (cited as *P. tortuosus*), *P. moreirai* Ingenito & Buckup, 2005 (cited as *Parodon* sp); Family Crenuchidae: *Characidium gomesi* Travassos, 1956, C. cf. zebra Eigenmann, 1909, *C. lauroi* Travassos, 1949, C. cf. alipioi Travassos, 1955 – UFSCar / PPGGEv / CNPq / CAPES

**V.11. MSc Dissertation by Caroline Garcia (2005)**: Contribuições aos estudos citogenéticos em algumas espécies de cinco famílias de Siluriformes do rio São Francisco / Contributions to cytogenetics of some species of three Siluriformes families from the São Francisco River. **Taxa analyzed**: Order Siluriformes – Family Auchenipteridae: *Trachelyopterus galeatus* (cited as *Paurachenipterus galeatus*), *T. leopardinus* (Borodin, 1927) cited as *Paurachenipterus leopardinus* (Borodin, 1927); Family Doradidae: *Fransciscodoras marmoratus* (Lütken, 1874); Family Heptapteridae: *Rhamdia quelen*; Family Pimelodidae: *Pimelodus fur* (Lütken, 1874), *P. maculatus* Lacepède, 1803, *Pimelodus* sp., *Zungaru zungaru* (Humboldt, 1821) cited as *Pseudopimelodus zungaru* (Humboldt, 1821) – UFSCar / PPGGEv / FAPESP

**V.12. PhD Thesis by Marcelo Ricardo Vicari (2006)**: Diversidade de peixes residentes em cabeceiras de rios. Uma abordagem cromossômica em três diferentes biomas aquáticos da região Sul do Brasil / Fish diversity from river headwaters. A chromosomal approach in three biomes from South Brazil. **Taxa analyzed**: Order Siluriformes – Family Callichthyidae: *Corydoras paleatus* (Jenyns, 1842), *C. ehrhardti* Steindachner, 1910; Order Characiformes – Family Parodontidae: *Apareiodon* sp.; Family Characidae: *Astyanax scabripinnis*, *A. janeiroensis* Eigenmann, 1908; Family Crenuchidae: Characidium cf. gomesi; Order Perciformes – Family Cichlidae: *Geophagus brasiliensis*, *Australoheros facetus* (Jenyns, 1842) cited as *Cichlasoma facetum* (Jenyns, 1842) – UFSCar / PPGGEv / FAPESP

**V.13. MSc Dissertation by Maressa Ferreira Neto (2008)**: Análise citogenética em algumas espécies de peixes de uma região divisora de águas entre riachos de bacias hidrográficas distintas / Cytogenetic analysis in fish species from a dividing water region of streams belonging to different river basins. **Taxa analyzed**: Order Characiformes – Family Characidae: *Astyanax altiparanae*, *A. fasciatus*, *Moenkausia sancta filomenae*; Family Curimatidae: *Cyphocarax modestus*; Family Prochilodontidae: *Prochilodus lineatus*; Order Gymnotiformes – Family Gymnotidae: *Gymnotus carapo* Linnaeus, 1758; Family Sternopygidae: *Eigenmannia* sp.; Order Perciformes – Family Cichlidae: *Geophagus brasiliensis* – UFSCar / PPGGEv / CAPES / CNPq

**V.14- PhD Thesis by Elisangela Bellafronte da Silva (2009)**: Citogenética clássica e molecular em peixes Neotropicais. Estudos comparativos entre bacias hidrográficas com ênfase em região de transposição de rio / Conventional and molecular cytogenetics in Neotropical fishes. Comparative studies among river basins with emphasis on a river transposition region. **Taxa analyzed**: Order Gymnotiformes – Family Gymnotidae: *Gymnotus carapo*, *G. silvius* Albert & Fernandes-Matioli, 1999; Family Sternopygidae: *Eigenmannia virescens* (Valenciennes, 1836), *Eigenmania* sp. – UFSCar / PPGGEv / CNPq / CAPES

**V.15. PhD Thesis by Daniel Luis Zanella Kantek (2010)**: Citogenética de espécies de Siluriformes da região de transposição do rio Piumhi (MG) / Cytogenetics of Siluriformes species from the transposition region of the Piumhi River (MG). **Taxa analyzed**: Order Siluriformes – Family Auchenipteridae: *Trachelyopterus galeatus* (cited as *Parauchenipterus galeatus*); Family Pimelodidae: *Pimelodus pohli* Ribeiro & Lucena, 2006; Family Heptapteridae: *Imparfinis schubarti* (Gomes, 1956); *Cetopsorhamdia iheringi* Schubart & Gomes, 1959, *Pimelodella vittata* (Lütken, 1874), *Rhamdia* sp. A, *Rhamdia* sp. B, Rhamdiopsis cf. microcephala (Lütken, 1874), Family Trichomycteridae: *Trichomycterus brasiliensis* Lütken, 1874 – UFSCar / PPGGEv / CNPq / CAPES

### Final remarks

Two general trends were found among the Neotropical fishes regarding the karyotype evolution. In fact, a significant number of families were characterized by conservative karyotypes, in contrast to others with highly divergent ones. Parodontidae, Anostomidae and Prochilodontidae species, for example, exhibit relatively homogeneous karyotypes at the macrostructural level, contrasting with the high chromosomal diversity found among Erythrinidae and Characidae species (Bertollo et al. 1986). It is noteworthy that karyotype features appear to be correlated with their lifestyle and ecological habits, since more dispersive and migratory species usually disclose more stable karyotypes when compared to those with low vagility and organized in small local populations (Bertollo et al. 1986; Blanco et al.2011; Oliveira et al. 2015). Indeed, many local populations were evidenced as having particular karyotypes, pointing to a large number of species complexes and the cryptic biodiversity present in the Neotropical fish fauna, as especially highlighted in the Characidae and Erythrinidae families (Moreira-Filho and Bertollo1991; Bertollo 2007; Cioffi et al. 2012a). In fact, many sympatric, or even syntopic, karyomorphs do not indicate hybridization at the chromosomal level, indicating the absence of gene flow among them and, consequently, corroborating the status of species complexes for some current nominal species (Bertollo et al. 2000).

Reports on chromosomal polymorphisms (Giuliano-Caetano and Bertollo 1988; Vicari et al. 2003; Pazza et al. 2006, 2008; Mariotto et al. 2009), natural triploidy (Morelli et al. 1983; Venere and Galetti Jr. 1985; Giuliano-Caetano and Bertollo 1990; Centofante et al. 2001; Garcia et al. 2003) and broad karyotype evolution by centric fissions (Feldberg et al. 1993), were also emphasized for distinct fish groups. Noteworthy is also the cytogenetic contribution for biogeographical analyzes, clarifying the current fish fauna distribution in some important Brazilian river basins. In this sense, native species, as well as invasive ones due to dispersal events or breakdown of geographic isolation, were clearly identified by chromosomal investigations (Peres et al. 2009; Blanco et al. 2010; Silva et al. 2010; Perez et al. 2012). As a significant example, *Astyanax bimaculatus* from two important Brazilian watersheds, namely the São Francisco and Grande rivers share similar morphological characteristics. However, specimens from each one of such rivers were well characterized by their particular chromosomal features. In the early 1960s, a tributary of the Grande River was artificially transposed into the São Francisco river basin, with the consequent breakdown of the geographic isolation of their respective fish fauna. As a consequence, cytogenetic investigation was able to identify representatives of *A. bimaculatus* from both basins living in sympatry in the transposition region, as well as individuals with intermediate karyotypes in view of the resulting secondary hybrid zone in such region (Peres et al. 2012).

Over the years, a particular emphasis has been directed on the characterization and the evolutionary process of sex chromosomes. A larger number of Neotropical fish species with well differentiated sex chromosomes occur in comparison to other world regions (Moreira-Filho et al. 1993), carrying simple (ZZ/ZW, XX/XY) and multiple (X_1_X_1_X_2_X_2_/X_1_X_2_Y, XX/XY_1_Y_2_, ZZ/ZW_1_W_2_) sex chromosome systems (Centofante et al. 2002; Cioffi et al. 2012b), in addition to some others disclosing a nascent or early stage of differentiation (Cioffi and Bertollo 2010; Freitas et al. in press). Usually, sex chromosomes occur as a particular feature for some species within a specific fish group, as exemplified in the Erythrinidae, Parodontidae, Anostomidae and Crenuchidae families (Galetti Jr. et al. 1981, 1995; Moreira-Filho et al. 1985, 1993; Molina et al. 1998; Centofante et al. 2001, 2003; Bertollo et al. 2000; Bertollo 2007; Vicari et al. 2008; Cioffi et al. 2013). As a singular exception, all species of the *Triportheus* genus (Triportheidae) share a same ZZ/ZW sex chromosome system (Artoni et al. 2001, 2002; Diniz et al. 2008), constituting a special model to investigate the evolution of the sex chromosomes among lower vertebrates. The modern molecular cytogenetics was a key step for understanding the evolutionary process of the sex chromosomes among fishes. This way, the significative role of several classes of repetitive DNAs in the differentiation path of the sex pair, both at its inicial stage (Cioffi and Bertollo 2010; Freitas et al. in press) or more advanced ones (Cioffi et al. 2010, 2011a, b, 2012b; Yano et al. 2014a, b), was clearly highlighted. Notably, whole chromosome painting (WCP) and comparative genomic hybridization (CGH) were able to demonstrate that fish sex chromosomes can have an independent origin even among closely related species (Cioffi et al. 2011c, d; 2013) or, alternatively, a common origin within particular monophyletic groups (Yano et al. 2016).

Besides sex chromosomes, supernumerary or B chromosomes comprise another special feature that stands out in the Neotropical fishes. Such additional elements can be i) as large as the biggest chromosome pair of the karyotype, ii) medium-sized, iii) very small iv) or even characterized as microchromosomes. Two particular models, represented by *Astyanax scabripinnis* and *Prochilodus lineatus*, have been subjected to continuous analyses over years. *A. scabripinnis* has some morphologically differentiated B chromosomes, although a large and similar in size to the first chromosome pair of the karyotype is the most frequent one (Moreira-Filho et al. 2004). Its origin as an isochromosome was demonstrated by both standard and molecular cytogenetic, including meiotic data (Vicente et al. 1996; Mestriner et al. 2000). A continuous population analysis showed that Bs display a particular dynamism related to environmental and sex conditions in *A. scabripinnis*. Indeed, it is noteworthy their gradual decrease in frequency from higher to lower altitudes, until the complete absence in the latter ones (Néo et al. 2000). In addition, an evident sex ratio distortion is associated with these chromosomes. In fact, the mean number of Bs in males is only about 27% of the female one, which matches the male population frequency (Vicente et al. 1996), suggesting that B chromosomes may play a role on sex determination in this species.

Constrasting with *A. scabripinnis*, *P. lineatus* bears a number of very small B chromosomes (Pauls and Bertollo 1983), which also have an intraspecific origin as indicated by molecular cytogenetic and chromosomal banding (Jesus et al. 2003; Artoni et al. 2006). Remarkably, the frequency of these chromosomes was changed over years in close association with their transmission dynamics. In this sense, the average number of Bs increased twice along a time period indicating an accumulation mechanism, but without evidences of additional changes after that. Significantly, the mitotic instability of Bs declined almost 400 times during this same period, reaching a stable transmission. This way, it is likely that the mitotic stabilization was a key process for neutralizing the accumulation process (Cavallaro et al. 2000).

Nowadays, many of such issues so far investigated, in addition to additional approaches on fish biology, are going in advancing in the light of chromosomal, cytogenomic and molecular methodologies currently available. It is hoped that these procedures can provide additional and important advances for the Neotropical fish fauna evolutionary history.

## References

[B1] Artoni RF Bertollo LAC (2002) Evolutinary aspects of the ZZ/ZW sex chromosome system in the Characidae fish, genus *Triportheus*. A monophyletic state and NOR location on the W chromosome. Heredity 89: 15–19. https://doi.org/10.1038/sj.hdy.6800081 12080365 10.1038/sj.hdy.6800081

[B2] Artoni RF Neves JNF Moreira-Filho O Bertollo LAC (2001) An uncommon condition for a sex chromosome system in Characidae fish. Distribution and differentiation of the ZZ/ZW system in *Triportheus*. Chromosome Research 9: 449–456. https://doi.org/10.1023/A:1011620226348 11592479 10.1023/a:1011620226348

[B3] Artoni RF Vicari MR Endler AL Cavallaro ZI Jesus CM Almeida MC Moreira-Filho O Bertollo LAC (2006) Banding pattern of A and B chromosomes of *Prochilodus lineatus* (Characiformes, Prochilodontidae), with comments on B chromosomes evolution. Genetica 127: 277–284. https://doi.org/10.1007/s10709-005-4846-1 16850231 10.1007/s10709-005-4846-1

[B4] Bertollo LAC (2007) Chromosome evolution in the Neotropical Erythrinidae fish family: an overview. In: Pisano E, Ozouf-Costaz C, Foresti F, Kapoor BG (Org) Fish Cytogenetics. 1ed. Enfield, New Hampshire, Science Publishers, 195–211.

[B5] Bertollo LAC Moreira-Filho O Galetti Jr PM (1986) Cytogenetics and taxonomy: considerations based on chromosome studies of freshwater fish. Journal of Fish Biology 28: 153–159.https://doi.org/10.1111/j.1095-8649.1986.tb05153.x

[B6] Bertollo LAC Cioffi MB Moreira-Filho O (2015) Direct chromosome preparation from freshwater teleost fishes. In: Ozouf-Costaz C, Pisano E, Foresti F, Almeida Toledo LF (Org) Fish Cytogenetic Techniques (Chondrichthyans and Teleosts). 1ed. CRC Press, Enfield, Boca Raton, 21–26. https://doi.org/10.1201/b18534-4

[B7] Bertollo LAC Born GG Dergam JA Fenocchio AS Moreira-Filho O (2000) A biodiversity approach in the Neotropical fish, *Hoplias malabaricus*. Karyotypic survey, geographic distribution of cytotypes and cytotaxonomic considerations. Chromosome Research 8: 603–613. https://doi.org/10.1023/A:1009233907558 11117356 10.1023/a:1009233907558

[B8] Blanco DR Lui RL Bertollo LAC Diniz D Moreira-Filho O (2010) Characterization of invasive fish species in a river transposition river: evolutionary chromosome studies in the genus *Hoplias* (Characiformes, Erythrinidae). Reviews in Fish Biology and Fisheries 20: 1–8. https://doi.org/10.1007/s11160-009-9116-3

[B9] Blanco DR Lui RL Vicari MR Bertollo LAC Moreira-Filho O (2011) Comparative cytogenetics of giant trahiras *Hoplias aimara* and *H. intermedius* (Characiformes, Erythrinidae): Chromosomal characteristics of minor and major ribosomal DNA and cross-species repetitive centromeric sequences mapping differ among morphologically identical karyotypes. Cytogenetics and Genome Research 132: 71–78. https://doi.org/10.1159/000320923 20924165 10.1159/000320923

[B10] Cavallaro ZI Bertollo LAC Perfectti F Camacho JPM (2000) Frequency increase and mitotic stabilization of B chromosomes in the fish *Prochilodus lineatus*. Chromosome Research 8: 627–634. https://doi.org/10.1023/A:1009242209375 11117359 10.1023/a:1009242209375

[B11] Centofante L Bertollo LAC Moreira-Filho O (2001) Comparative cytogenetics among sympatric species of *Characidium* (Pisces, Characiformes). Diversity analysis with the description of a ZZ/ZW sex chromosome system and natural triploidy. Caryologia 54: 253–260. https://doi.org/10.1080/00087114.2001.10589233

[B12] Centofante L Bertollo LAC Moreira-Filho O (2002) A ZZ/ZW sex chromosome system in a new species of the genus *Parodon* (Pisces, Parodontidae). Caryologia 55: 139–150. https://doi.org/10.1080/00087114.2002.10589270

[B13] Centofante L Bertollo LAC Buckup PA Moreira-Filho O (2003) Chromosomal divergence and maintenance of sympatric *Characidium* species (Crenuchidae, Characidiinae). Hereditas 138: 213–218. https://doi.org/10.1034/j.1601-5223.2003.01714.x 14641486 10.1034/j.1601-5223.2003.01714.x

[B14] Cioffi MB Bertollo LAC (2010) Initial steps in XY chromosome differentiation in *Hoplias malabaricus* and the origin of an X1X2Y sex chromosome system in this fish group. Heredity 105: 554–561. https://doi.org/10.1038/hdy.2010.18 20216570 10.1038/hdy.2010.18

[B15] Cioffi MB Martins C Vicari MR Rebordinos L Bertollo LAC (2010) Differentiation of the XY sex chromosomes in the fish *Hoplias malabaricus* (Characiformes, Erythrinidae): Unusual accumulation of repetitive sequences on the X chromosome. Sexual Development 4: 176–185. https://doi.org/10.1159/000309726 20502069 10.1159/000309726

[B16] Cioffi MB Camacho JPM Bertollo LAC (2011a) Repetitive DNAs and differentiation of sex chromosomes in Neotropical fishes. Cytogenetic and Genome Research 132: 188–194. https://doi.org/10.1159/000321571 21042005 10.1159/000321571

[B17] Cioffi MB Kejnovsky E Bertollo LAC (2011b) The chromosomal distribution of microsatellite repeats in the wolf fish genome *Hoplias malabaricus*, focusing on the sex chromosomes. Cytogenetic and Genome Research 132: 289–296. https://doi.org/10.1159/000322058 21099206 10.1159/000322058

[B18] Cioffi MB Sánchez A Marchal JA Kosyakova N Liehr R Trifonov V Bertollo LAC (2011c) Cross-species chromosome painting tracks the independent origin of multiple sex chromosomes in two cofamiliar Erythrinidae fishes. BMC Evolutionary Biology 11: 186. https://doi.org/10.1186/1471-2148-11-186 10.1186/1471-2148-11-186 PMC3141436 21718509

[B19] Cioffi MB Sánchez A Marchal JA Kosyakova N Liehr T Trifonov V Bertollo LAC (2011d) Whole chromosome painting reveals independent origin of sex chromosomes in closely related forms of a fish species. Genetica 139: 1065–1072. https://doi.org/10.1007/s10709-011-9610-0 21927842 10.1007/s10709-011-9610-0

[B20] Cioffi MB Molina WF Artoni RF Bertollo LAC (2012a) Chromosomes as tools for discoreving biodiversity.The case of Erythrinidae fish family. In: Tirunilai P (Org) Recent Trends in Cytogenetic Studies. Methodologies and Applications. Intech, Rijeka, 125–146.

[B21] Cioffi MB Moreira-Filho O Almeida-Toledo LF Bertollo LAC (2012b) The contrasting role of heterochromatin in the differentiation of sex chromosomes: an overview from Neotropical fishes. Journal of Fish Biology 80: 2125–2139. https://doi.org/10.1111/j.1095-8649.2012.03272.x 22551173 10.1111/j.1095-8649.2012.03272.x

[B22] Cioffi MB Liehr T Trifonov V Molina WF Bertollo LAC (2013) Independent sex chromosome evolution in lower vertebrates: A molecular cytogenetic overview in the Erythrinidae fish family. Cytogenetic and Genome Research 141: 186–194. https://doi.org/10.1159/000354039 23919986 10.1159/000354039

[B23] Diniz D Laudicin A Cioffi MB Bertollo LAC (2008) Microdissection and whole chromosome painting. Improving sex chromosome analysis in *Triportheus* (Teleostei, Characiformes). Cytogenetic and Genome Research 122: 163–168. https://doi.org/10.1159/000163094 19096212 10.1159/000163094

[B24] Feldberg E Porto JIR Bertollo LAC Nakayama C (1993) Karyotype evolution in Curimatidae (Teleostei, Characiformes) from the Amazon region. II. Centric fissions in the genus *Potamorhina*. Genome 36: 372–376. https://doi.org/10.1139/g93-051 18469994 10.1139/g93-051

[B25] FishBase: www.fishbase.org, version 10/2016.

[B26] Freitas NL Al-Rikabi ABH Bertollo LAC Ezaz T Yano CF Oliveira EA Hatanaka T Cioffi MB (in press) A cryptically differentiated XX/XY sex chromosome system in the fish *Hoplias malabaricus* (Characiformes, Erythrinidae) revealed by DNA repeats accumulation on the young Y chromosome. Current Genomic. 10.2174/1389202918666170711160528 PMC5850510 29606909

[B27] Galetti Jr PM Foresti F Bertollo LAC Moreira-Filho O (1981) Heteromorphic sex chromosomes in three species of the genus *Leporinus* (Pisces, Anostomidae). Cytogenetics and Cell Genetics 29: 138–142. https://doi.org/10.1159/000131562 7226896 10.1159/000131562

[B28] Galetti Jr PM Lima NRW Venere PC (1995) A monophyletic ZW sex chromosome system in *Leporinus* (Anostomidae, Characiformes). Cytologia 60: 375–382. https://doi.org/10.1508/cytologia.60.375

[B29] Garcia C Moreira-Filho O Bertollo LAC Centofante L (2003) B chromosomes and natural triploidy in *Rhamdia* sp (Pisces, Siluriformes, Heptapteridae). Cytologia 68: 403–411. https://doi.org/10.1508/cytologia.68.403

[B30] Giuliano-Caetano L Bertollo LAC (1988) Karyotype variability in *Hoplerythrinus unitaeniatus* (Characiformes, Erythrinidae). I. Chromosome polymorphism in the Rio Negro population (Manaus, State of Amazonas). Brazilian Journal of Genetics 11: 299–306.

[B31] Giuliano-Caetano L Bertollo LAC (1990) Karyotypic variability in *Hoplerythrinus unitaeniatus* (Pisces, Characiformes, Erythrinidae). II. Occurrence of natural triploidy. Brazilian Journal of Genetics 13: 231–237.

[B32] Jesus CM Galetti Jr PM Valentini SR Moreira-Filho O (2003) Molecular characterization and chromosomal localization of two families of satellite DNA in *Prochilodus lineatus* (Pisces, Prochilodontidae), a species with B chromosomes. Genetica 118(1): 25–32. https://doi.org/10.1023/A:1022986816648 12737143 10.1023/a:1022986816648

[B33] Mariotto S Centofante L Miyasawa CS Bertollo LAC Moreira-Filho O (2009) Chromosome polymorphism in *Ancistrus cuiabae* Knaack, 1999 (Siluriformes: Loricariidae: Ancistrini). Neotropical Ichthyology 7: 595–600. https://doi.org/10.1590/S1679-62252009000400006

[B34] Mestriner CA Galetti Jr PM Valentini SR Ruiz IRG Abel LDS Moreira-Filho O Camacho JPM (2000) Structural and functional evidence that a B chromosome in the characid fish *Astyanax scabripinnis* is an isochromosome. Heredity 85: 1–9. https://doi.org/10.1046/j.1365-2540.2000.00702.x 10971685 10.1046/j.1365-2540.2000.00702.x

[B35] Molina WF Schmid M Galetti Jr PM (1998) Heterochromatin and sex chromosomes in the Neotropical fish genus *Leporinus* (Characiformes, Anostomidae). Cytobios 94: 141–149.

[B36] Moreira-Filho O Bertollo LAC (1991) *Astyanax scabripinnis* (Pisces, Characidae): a species complex. Brazilian Journal of Genetics 14: 331–357.

[B37] Moreira-Filho O Bertollo LAC Galetti Jr PM (1985) Karyotypic studies of some species of the family Parodontidae (Pisces, Cypriniformes). Caryologia 38: 47–55. https://doi.org/10.1080/00087114.1993.10797253

[B38] Moreira-Filho O Bertollo LAC Galetti Jr PM (1993) Distribution of sex chromosome mechanisms in Neotropical fish and description of a ZZ/ZW system in *Parodon hilarii* (Parodontidae). Caryologia 46: 115–125. https://doi.org/10.1080/00087114.1993.10797253

[B39] Moreira-Filho O Galetti Jr PM Bertollo LAC (2004) B chromosomes in the fish *Astyanax scabripinnis* (Characidae, Tetragonopterinae): an overview in natural populations. Cytogenetic and Genome Research 106: 230–234. https://doi.org/10.1159/000079292 15292596 10.1159/000079292

[B40] Morelli S Bertollo LAC Moreira-Filho O (1983) Cytogenetics considerations on the genus *Astyanax* (Pisces, Characidae). II. Occurrence of natural triploidy. Caryologia 36: 245–250. https://doi.org/10.1080/00087114.1983.10797665

[B41] Néo DM Bertollo LAC Moreira-Filho O Camacho JPM (2000) Altitudinal variation for B chromosome frequency in the characid fish *Astyanax scabripinnis*. Heredity 85: 136–141. https://doi.org/10.1046/j.1365-2540.2000.00744.x 11012715 10.1046/j.1365-2540.2000.00744.x

[B42] Oliveira C Avelino GS AbeK T Mariguela TC Benine RC Orti G Vari RP Correa e Castro RM (2011) Phylogenetic relationships within the speciose family Characidae (Teleostei: Ostariophysi: Characiformes) based on multilocus analysis and extensive ingroup sampling. BMC Evolutionary Biology 11: 275. https://doi.org/10.1186/1471-2148-11-275 10.1186/1471-2148-11-275 PMC3190395 21943181

[B43] Oliveira EA Bertollo LAC Yano CF Liehr T Cioffi MB (2015) Comparative cytogenetics in the genus *Hoplias* (Characiformes, Erythrinidae) highlights contrasting karyotype evolution among congeneric species. Molecular Cytogenetics 8: 56. https://doi.org/10.1186/s13039-015-0161-4 10.1186/s13039-015-0161-4 PMC4518567 26225139

[B44] Pauls E Bertollo LAC (1983) Evidence for a system of supernumerary chromosomes in *Prochilodus scrofa* Steindachner, 1881 (Pisces, Prochilodontidae). Caryologia 36: 307–314. https://doi.org/10.1080/00087114.1983.10797671

[B45] Pazza R Kavalco KF Bertollo LAC (2006) Chromosome polymorphism in *Astyanax fasciatus* (Teleostei, Characiformes). 1- Karyotypic analysis, Ag-NORs and mapping of the 18S and 5S ribosomal genes in sympatric karyotypes and their possible hybrid forms. Cytogenetic and Genome Research 112: 313–319. https://doi.org/10.1159/000089886 16484788 10.1159/000089886

[B46] Pazza R Kavalco KF Bertollo LAC (2008) Chromosome polymorphism in *Astyanax fasciatus* (Teleostei, Characidae). 2. Chromosomal location of a satellite DNA. Cytogenetic and Genome Research 122: 61–66. https://doi.org/10.1159/000151317 18931487 10.1159/000151317

[B47] Peres WAM Buckup PA Kantek DL Bertollo LAC Moreira-Filho O (2009) Chromosomal evidence of downstream dispersal of *Astyanax fasciatus* (Characiformes, Characidae) associated with river shed interconnection. Genetica 137: 305–311. https://doi.org/10.1007/s10709-009-9389-4 19641999 10.1007/s10709-009-9389-4

[B48] Peres WAM Bertollo LAC Buckup PA Blanco DR Kantek DLZ Moreira-Filho O (2012) Invasion, dispersion and hybridization of fish associated to river transposition: karyotypic evidence in *Astyanax*. Reviews in Fish Biology and Fisheries 22: 519–526. https://doi.org/10.1007/s11160-011-9246-2

[B49] Reis RE Kullander SO Ferraris Jr CJ (2003) Check list of freshwater fishes of South and Central America. EDIPUCRS, Porto Alegre, 742 pp.

[B50] Silva EB Moreira-Filho O Vicari MR Artoni RF Bertollo LAC Margarido VP (2010) Cytogenetic identification of invasive fish species following connections between hydrographic basins. Hydrobiologia 649: 347–354. https://doi.org/10.1007/s10750-010-0277-9

[B51] Venere PC Galetti Jr PM (1985) Natural triploidy and chromosomes B in the fish *Curimata modesta* (Curimatidae, Characiformes). Brazilian Journal of Genetics 4: 681–687.

[B52] Vicari MR Artoni RF Bertollo LAC (2003) Heterochromatin polymorphism associated with 18S rDNA: a differential pathway among *Hoplias malabaricus* fish populations. Cytogenetic and Genome Research 101: 24–28. https://doi.org/10.1159/000073413 14571132 10.1159/000073413

[B53] Vicari MR Artoni RF Moreira-Filho O Bertollo LAC (2008) Diversification of a ZZ/ZW sex chromosome system in *Characidium* fish (Crenuchidae, Characiformes). Genetica 134: 311–317. https://doi.org/10.1007/s10709-007-9238-2 18175199 10.1007/s10709-007-9238-2

[B54] Vicente VE Moreira-Filho O Camacho JPM (1996) Sex-ratio distortion associated with the presence of a B chromosome in *Astyanax scabripinnis* (Teleostei, Characidae). Cytogenetics and Cell Genetics 74: 70–75. https://doi.org/10.1159/000134385 8893805 10.1159/000134385

[B55] Yano CF Bertollo LAC Molina WF Liehr T Cioffi MB (2014a) Genomic organization of repetitive DNAs and its implications for male karyotype and the neo-Y chromosome differentiation in *Erythrinus erythrinus* (Characiformes, Erythrinidae). Comparative Cytogenetics 8: 139–151. https://doi.org/10.3897/compcytogen.v8i2.7597 25147625 10.3897/CompCytogen.v8i2.7597 PMC4137284

[B56] Yano CF Poltronieri J Bertollo LAC Artoni RF Liehr T Cioffi MB (2014b) Chromosomal mapping of repetitive DNAs in *Triportheus trifurcatus* (Characidae, Characiformes): Insights into the differentiation of the Z and W chromosomes. Plos One 9: e90946. https://doi.org/10.1371/journal.pone.0090946 10.1371/journal.pone.0090946 PMC3954618 24632562

[B57] Yano CF Bertollo LAC Ezaz T Trifonov V Sember A Liehr T Cioffi MB (2016) Highly conserved Z and molecularly diverged W chromosomes in the fish genus *Triportheus* (Characiformes, Triportheidae). Heredity 118: 276–283. https://doi.org/10.1038/hdy.2016.83 28000659 10.1038/hdy.2016.83 PMC5315523

